# Resection of a recurrent pineal region teratoma via a posterior interhemispheric transcallosal approach

**DOI:** 10.3171/2021.4.FOCVID2134

**Published:** 2021-07-01

**Authors:** David S. Hersh, Scott Boop, Frederick A. Boop

**Affiliations:** 1 Division of Neurosurgery, Connecticut Children’s, Hartford;; Departments of 2 Surgery and; 3 Pediatrics, UConn School of Medicine, Farmington, Connecticut;; 4 Department of Neurological Surgery, University of Washington, Seattle, Washington;; 5 Department of Neurosurgery, University of Tennessee Health Science Center, Memphis;; 6 Le Bonheur Children’s Hospital, Memphis; and; 7 Semmes-Murphey Clinic, Memphis, Tennessee

**Keywords:** pineal, transcallosal, interhemispheric, teratoma, mixed germ cell

## Abstract

The authors describe the unusual case of a 6-year-old boy presenting with decorticate posturing, diminished hearing, and an inability to open his eyes, despite being verbally responsive. He underwent a posterior interhemispheric transcallosal intervenous approach for resection of a pineal region mature teratoma, which recurred 2 years postoperatively. This video demonstrates his initial surgery and reresection, illustrating the value of this approach for more complex lesions that involve the internal cerebral veins (ICVs). At the time of recurrence, microsurgical dissection of the scarred interhemispheric fissure was required to facilitate removal of the multifocal recurrent teratoma, resulting in gross-total resection.

The video can be found here: https://stream.cadmore.media/r10.3171/2021.4.FOCVID2134.

## Transcript

In this video, we describe a posterior interhemispheric transcallosal approach for resection of a recurrent pineal region teratoma.

### 0:27 History and Exam

The patient was a 6-year-old male who initially presented to an outside hospital with headaches and was found to have a pineal region mass with obstructive hydrocephalus. He underwent an endoscopic biopsy and VP shunt placement, with subsequent placement of two additional Ommaya catheters into cystic components of the mass. The diagnosis was consistent with a mixed germ cell tumor, and the patient underwent two cycles of chemotherapy, but his exam declined and subsequent imaging demonstrated rapid enlargement of the mass. He was transferred for further management, and upon arrival he was noted to be opisthotonic, with decorticate posturing, inability to open his eyes, tonic gaze deviation to the left, and decreased hearing, though his pupils were equal and reactive and he was able to respond verbally.

### 1:08 Imaging

The patient’s initial imaging demonstrated a large, heterogeneous, contrast-enhancing mass with solid and cystic components exerting significant mass effect on the tectum and dorsal brainstem. Once stabilized, the patient was taken to the OR for resection of the mass.

### 1:22 Surgical Options

Three primary approaches to the pineal region and posterior third ventricle have been described—the supracerebellar infratentorial approach, the occipital interhemispheric transtentorial approach, and the posterior interhemispheric transcallosal approach, which can be performed utilizing intervenous or paravenous variants.^1–3^

### 1:39 Choosing an Approach

Each tumor in this region is unique, and choosing the optimal approach should take several considerations into account: the dimensions of the tumor, particularly in the anterior-posterior and rostral-caudal axes; proximity to other neurovascular structures such as the corpus callosum, midbrain, superior cerebellum, thalamus, and deep venous system; the angle of the tentorium; the consistency of the tumor; and the individual surgeon’s training, experience, and comfort level.^2^

### 2:03 Anatomy

For this particular tumor, the intervenous variant of the posterior interhemispheric transcallosal approach was chosen. Notably, there is a natural separation of the fornices posteriorly, where they diverge into the forniceal crura. The fornices are always lateral to the internal cerebral veins at this level. Therefore, by maintaining a plane of dissection between the two ICVs, the fornices remain protected while accessing the velum interpositum—the roof of the third ventricle.

### 2:28 Posterior Interhemispheric Transcallosal Approach

The posterior interhemispheric approach has a number of advantages, including wide access to the surrounding structures and early identification and control of the ICVs. This expanded corridor is particularly useful for mature teratomas, which have elements of bone, cartilage, and muscle that are not easily removed with an ultrasonic aspirator and which must often be removed piecemeal; this can be challenging when working in a deep space with a long, narrow corridor. Similarly, the resection of bloody tumors such as pineoblastomas may be facilitated by an expanded corridor, whereas PNETs and gliomas are often easily removed with an ultrasonic aspirator, and are amenable to a variety of surgical approaches.

The posterior interhemispheric approach is particularly useful in tumors with a long rostral-caudal axis, where the upper aspect of the tumor is immediately underneath the corpus callosum. Additionally, this approach avoids prone positioning and is relatively ergonomic, allowing two surgeons to work across from each other and utilize the four-hand technique. Disadvantages of the posterior interhemispheric approach include a potential blind spot underneath and behind the splenium of the corpus callosum, the need to sometimes sacrifice a cortical bridging vein, and the need for brain relaxation in order to avoid excessive retraction on the medial hemisphere, which can result in postoperative weakness.^2^

### 3:42 Positioning and Incision

In order to perform the posterior transcallosal approach, one option is to place the patient in a lateral position, with the ipsilateral hemisphere in the dependent position to facilitate retraction by gravity. However, we prefer to place the patient in the supine position with the neck flexed in order to orient the vertex to the ceiling. We find that this provides a natural orientation for the surgeon and facilitates involvement by the assistant surgeon using the four-hand technique. Once the patient was positioned, the midline was marked and frameless stereotactic navigation was used to identify trajectories to the anterior and posterior aspects of the tumor. A biparietal zigzag incision was then marked.

### 4:17 Exposure

The skin was incised and self-retaining retractors were placed. Burr holes were made on either side of the midline anteriorly and posteriorly, and a biparietal craniotomy was performed following dissection of the epidural space. Gelfoam was placed over the sagittal sinus, and the dura was opened on the right in a C-shaped fashion and flapped toward the midline.

### 4:34 Surgical Technique

The microscope was brought into the field. Sutures were placed in the falx and tied to epidural tack-up holes in the contralateral parietal bone, in order to mobilize the falx and expand the surgical corridor. Microsurgical dissection techniques were used to dissect the interhemispheric fissure down to the pericallosal vessels. Cottonoids were placed to protect the medial surface of the hemisphere. Fixed retractors were not used at all during the case. The anterior and posterior limits of the needed callosotomy were then defined with frameless stereotactic guidance, and the corpus callosum was opened to the right of midline.

Immediately upon coming through the thinned-out corpus callosum, the tumor capsule was encountered. The capsule was opened and the ultrasonic aspirator was used to begin to debulk the tumor, which appeared to be filled with thick mucinous tissue in some regions, whereas in other regions we encountered areas of cartilage, squamous epithelium, and various teratomatous elements. Firm areas of the tumor could not be removed effectively with the aspirator, and sharp dissection was required.

The tumor was noted to be adherent to the internal cerebral veins. The left ICV was identified, and sharp dissection was used to release it from the tumor, taking care not to injure the vessel itself. Similarly, the right ICV was identified, and sharp dissection was again used to release it from the tumor. Once the internal cerebral veins were identified, the ultrasonic aspirator was then used to debulk the tumor where possible.

Upon releasing the ICVs, we were able to complete our circumferential dissection of the tumor and remove it as one piece.

Upon removal of the tumor, CSF could be seen arising from the cerebral aqueduct, which appeared patent.

### 6:13 Closing

The wound was copiously irrigated and hemostasis was obtained. All hemostatic agents were removed from the resection cavity. A watertight dural closure was performed.

The bone flap was then replaced and secured with titanium plates and screws, and the galea and skin were closed in layers.

### 6:27 Postoperative Course

Pathology was consistent with a mixed germ cell tumor. The patient’s neurological exam improved quickly postoperatively. He was discharged home on postoperative day 8 and did not require further adjuvant treatment. However, a surveillance scan 2 years later demonstrated an asymptomatic recurrence of the tumor.

### 6:44 Imaging—Recurrence

Imaging demonstrated diffusion-restricting nodules at the site of the prior resection, in the region of the posterior third ventricle and aqueduct.

Contrast-enhanced imaging demonstrated one nodule of tumor to the right of midline, with another in the region of the aqueduct, and a third underlying the splenium.

Tumor markers were negative, suggesting that the recurrence was likely a mature teratoma.^4^ The patient was therefore taken back to the operating room for resection of the mass.

The patient was positioned in a similar fashion as during his original surgery. The incision was reopened and the biparietal bone flap was removed.

### 7:15 Surgical Technique—Recurrence

The dura was opened medial to the prior suture line; however, the pia was quite adherent to the dura, and we noted a small chronic subdural membrane. Therefore, the microscope was brought into the field, the dura was opened with an 11 blade, and microsurgical dissection was performed to separate the dura from the chronic subdural membrane.

At the midline, the membrane was opened and a pial dissection was performed to separate the pia from the interhemispheric fissure. The old callosotomy was identified and opened. This led us into the ventricle, where the first tumor nodule was identified overlying the aqueduct, consisting primarily of whorls of hair. The tumor seemed to be arising from the inferior aspect of the junction of the ICVs and the vein of Galen. The capsule of the tumor was bipolared and cut with microscissors, leaving the vein intact. Eventually, we were able to work around the tumor and hand it off the field, exposing the patent cerebral aqueduct. Gelfoam was placed over the aqueduct to prevent rundown of blood products.

Just above that area, there was a second nodule of tumor with a classic epidermoid, pearly appearance which was dissected out and removed.

Finally, image guidance was used to identify the third nodule of tumor near the splenium of the corpus callosum. This nodule had a tough, leathery capsule and appeared to be attached to the adjacent vein. The microbipolar was used to coagulate the capsule, and microscissors were used to transect it, staying just above the vein. Laterally, vessels entering the capsule from the choroid plexus in the atrium of the right lateral ventricle were coagulated and cut. Eventually, we were able to mobilize the whole encapsulated tumor and resect it away from the veins, leaving them intact. This was handed off as a separate specimen. The thick, leathery capsule was opened and sebaceous material was identified.

The cavity was inspected under the microscope, and anything suspicious for residual tumor was removed. There was good egress of clear fluid up from the aqueduct. The wound was copiously irrigated and hemostasis was obtained. All hemostatic agents were removed except for a small piece of Gelfoam on the vein of Galen.

### 9:51 Closing—Recurrence

A watertight dural closure was obtained with the incorporation of a bovine pericardial patch graft. The bone flap was resecured with titanium plates and screws, and the galea and skin were closed with absorbable sutures.

### 10:01 Postoperative Course—Recurrence

Postoperative imaging demonstrated a gross-total resection. Pathology was consistent with a mature teratoma. The patient’s neurological exam remained intact postoperatively, and he was discharged on postoperative day 4.

### 10:13 Follow-Up

At his most recent follow-up, 20 months after his second resection, he was noted to have an intact exam with full extraocular movements.

### 10:20 Follow-Up Imaging

Follow-up imaging at that time revealed postoperative changes without evidence of recurrence to date.

## Author Contributions

Primary surgeon: FA Boop. Editing and drafting the video and abstract: all authors. Critically revising the work: Hersh, S Boop. Reviewed submitted version of the work: all authors. Approved the final version of the work on behalf of all authors: Hersh. Supervision: FA Boop.

## Previous Presentations

A subset of this video involving the original resection was presented at the “Operative Nuances I: Tackling Challenging Cases 3D Video Presentation” session of the 84th AANS Annual Scientific Meeting, Chicago, Illinois, 2016.
